# Real-Time Vehicle Motion Detection and Motion Altering for Connected Vehicle: Algorithm Design and Practical Applications

**DOI:** 10.3390/s19194108

**Published:** 2019-09-23

**Authors:** Wei Zhao, Jiateng Yin, Xiaohan Wang, Jia Hu, Bozhao Qi, Troy Runge

**Affiliations:** 1 College of Agricultural and Life Sciences, University of Wisconsin-Madison, Madison, WI 53705, USA; 2 State Key Laboratory of Vehicle NVH and Safety Technology, Beijing 100044, China; 3 State Key Laboratory of Rail Traffic Control and Safety, Beijing Jiaotong University, Beijing 100044, China; 4 College of Transportation Engineering, Dalian Maritime University, Dalian 116026, China; 5 College of Transportation Engineering, Tongji University, Shanghai 200092, China; 6 Department of Electrical and Computer Engineering, University of Wisconsin-Madison, Madison, WI 53705, USA

**Keywords:** smartphone sensors, real-time motion detection, connected vehicle, IMU

## Abstract

Real-time capturing of vehicle motion is the foundation of connected vehicles (CV) and safe driving. This study develops a novel vehicle motion detection system (VMDS) that detects lane-change, turning, acceleration, and deceleration using mobile sensors, that is, global positioning system (GPS) and inertial ones in real-time. To capture a large amount of real-time vehicle state data from multiple sensors, we develop a dynamic time warping based algorithm combined with principal component analysis (PCA). Further, the designed algorithm is trained and evaluated on both urban roads and highway using an Android platform. The aim of the algorithm is to alert adjacent drivers, especially distracted drivers, of potential crash risks. Our evaluation results based on driving traces, covering over 4000 miles, conclude that VMDS is able to detect lane-change and turning with an average precision over 76% and speed, acceleration, and brake with an average precision over 91% under the given testing data dataset 1 and 4. Finally, the alerting tests are conducted with a simulator vehicle, estimating the effect of alerting back or front vehicle the surrounding vehicles’ motion. Nearly two seconds are gained for drivers to make a safe operation. As is expected, with the help of VMDS, distracted driving decreases and driving safety improves.

## 1. Introduction

Safety is a cornerstone of both connected vehicles and advanced driver assistance systems (ADAS). More complex safety problems are expected in mixed traffic of automated and manual vehicle in level three connected vehicles as a result of distraction [1]. Distracted driving is one of the most dangerous reasons increasing crash risk. Although people are aware of risks of sending messages or making phone calls while driving, they still fail to focus on driving all the time anyway. In 2014, 3179 people died, and 431,000 people were injured in traffic crashes associated with distracted driving [2]. Traffic risks can be intuitively understood as the probability and severity of accidents that drivers have a chance to encounter in the future. According to this concept, in order to decrease the risk in a particular driving scenario, it is necessary to create an accurate vehicle motion detection system (VMDS) that can detect driving status as well as share information with vehicles around it in real-time. With VMDS, a kind of ADAS, drivers are able to have more time and space to respond to the potential risk. It is reported that 80% of existing crashes could have been prevented if the risk could be sensed and drivers could communicate with each other [3].

Mobile phones have been applied for driving safety in recent research. In the past study, experimental vehicles with mobile phone sensors are used to record driving data or test if there exist potential crashes. Smartphone sensors’ data are commonly used to collect and evaluate the traffic prediction model and estimate the freeway traffic status [4]. A mobile device was also used to obtain vehicle location information and process received data. Then, the route information was shared with a mobile phone in order to test the traffic-monitoring system [5]. A study [6] developed a technique to detect the driving behavior of drunk drivers. Driving performance was used by the proposed system to judge whether the drivers were intoxicated. The authors of [7] also collected driving motion data using sensors embedded in mobile phones. A two-stage clustering approach is developed for detecting dangers events, acceleration profile, and speeding. An experiment with a larger number of samples including 100 drivers’ inertial measurement unit (IMU) sensor data was explained in the work of [8]. This research took advantage of both smartphone sensors and the back-end server, exploring the performance of the model on predicting phone usage in different road-types/length trips. Furthermore, The authors of [9,10] argued the existing drawbacks of using smartphone sensors inters of sensors hardware, experiment methods, and data analytic algorithms. Cybernetics model and machine learning methods were applied to enhance the capability of precise driving monitoring. 

Vehicle motion detection is always the focus of transportation research. Most of the conceptive methods are based on high-end sensors. Multiple external sensors, like microphone, accelerometer, and radio, are demonstrated to detect the motion and status of traffic. The driving status associated with crash is analyzed with real-time trip data to recognize a potential accident [11]. Microsoft has designed a system to detect traffic honking, road bumps, and brakes with external sensors [12]. With the help of external accelerometers, others studies [13,14] developed a pothole patrol system for detecting road conditions, for example, recognizing bumpy road surfaces. The authors of [15] focus on driving activity detection and driving events recognition via addressing a new approach to optimize the data window size and overlapping ratio for every single vehicle for training model purposes.

### 1.1. Lane Changing and Turning Detection

LaneQuest system was composed and compared with other lane estimation techniques using mobile phones [16]. An iPhone camera was proposed to detect the lane markings [17]. A study [18] illustrated that with the assistance of the multicore technique of developed smartphones, the lane-change detection accuracy would be promoted. Cameras have been widely used to detect lane changes and lane keeping. However, inaccurate detection results are susceptible to errors under certain scenarios, such as poor light conditions and adverse weather.

Turning, especially left turning at uncontrolled intersections or ramps, is one of the most serious safety concerns. Turning detection is conducted in the situation that the left-turning vehicle is confronted by oncoming traffic [19]. Twenty-seven percent of crossing road accidents happened while vehicles were making a turning in the United States. Detailed research showed that it would take 4.1 s on average for a vehicle to turn left at a rural T-intersection. Further, it would take 4.0 and 4.3 s on average at two intersections in Pennsylvania [20]. Currently, there are two common methods for lane change detection. One common lane change detection method predicts vehicle trajectories and uses a list of rules to monitor the driver’s maneuvers. Hidden Markov model (HMM) is also used to detect and estimate driver actions [21,22]. The authors of [23] built an HMM model for driver behavior monitoring and improving driving safety based on detected driver behaviors. The other detection method is based on emerging artificial intelligence techniques. The authors of Another paper [24] uses support vector machine (SVM) to detect lane change events around the ego vehicles. A further study [25] designed a surrounding vehicle lane changing model using deep learning neural networks. Neural networks are becoming more and more popular as they can be a combination of both linear and non-linear network functions. However, training an effective neural network requires a representative and considerable dataset, as well as powerful computing machines. Furthermore, the result analysis process can be complex because most of the neural networks are black boxes.

### 1.2. Detection of Acceleration and Deceleration/Brakes

Various techniques and methods of detecting overtaking have been researched. The works of [26,27] have promoted a system that used GPS and phones to detect acceleration and deceleration to estimate the congestion. A mixed algorithm was created to detect the acceleration, combining dynamic planning with robust information [28]. A wireless sensor networks layout was designed to monitor vehicles [29]. Moreover, the future motion was predicted using dynamic and kinematic models making certain control inputs, vehicle capabilities, and the external situation related to the updating status of vehicles [30]. 

Any connected safety applications are less meaningful unless they are widely applied in most vehicles. However, full-scaled vehicle motion detection is a challenging task and a long-term issue in mixed traffic of automated and manual vehicles. First, there is hardly a common standard device that was approved as an accurate detector. Second, as a result of diverse car manufactories and cost of communication devices, it will take a long time to come to an agreement on the popularization of the same model device. For example, the most common devices are loop detectors, magnetic sensors, acoustic sensors, and computer vision techniques. However, these techniques require special hardware to be installed either on the infrastructures or in vehicles. This also limits the wide application and scalability because of the high cost [31].

Therefore, as this study proposes, smartphones, which can be utilized as both sensors detecting the vehicle motions and communication devices, are selected as the most common safety applications in levels 2 and 3 connected vehicles [32]. More specifically, this study develops a real-time-capable traffic and ego vehicle motion detection system, which is based on a dynamic time warping based algorithm combined with principal component analysis (PCA). This system can detect the motion of the self-vehicle, and then the motion information is able to transmit to the back-and-front vehicles. Thus, the drivers in the adjacent vehicle are alerted and can notice the potential risks earlier. With popularization and accuracy as our motivation, a cheap and convenient mobile device and a customized Android application are utilized to analyze and advise the driver on sudden and harmful situations that arise from self-vehicle and surrounding traffic factors.

**Figure 1:**
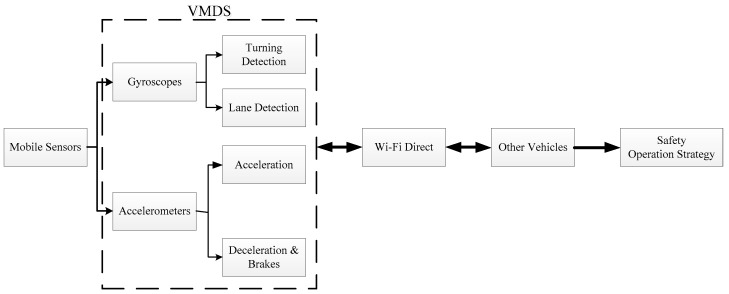
Overview vision for the vehicle motion detection system (VMDS) and outlines of this study.

Figure 1 gives an overview of the vision for the VMDS. The system consists of two parts: (1) motion detection with mobile sensors, such as turning, lane-changing, acceleration, and brakes; (2) transmitting this motion detection information to others by Wi-Fi Direct.

Our contribution in this paper is specified as follows.

- Real-timely detect lane changing, turning (curve and intersection), and acceleration using inertial measurement unit (IMU) sensors-gyroscopes embedded in smartphones. A dynamic time warping based algorithm combined with PCA is proposed as the core algorithm for this purpose.
- Real-time detection is applied to capture vehicle motion parameters in terms of acceleration, deceleration, and brakes using accelerometers embedded in mobile phones. Accelerometers can be a good candidate to estimate such vehicle motions.
- Develop a real-time vehicle motion detection system (VMDS), which can detect the ego vehicle’s motion and share the motion information with front-and-back vehicles, and even alert the drivers in front-and-back vehicles of the motion via Wi-Fi Direct. The use of VMDS is practiced and evaluated in a real test-bed based on Android phones.
- Using a driving simulator to evaluate how much time was gained for the drivers in the adjacent front-or-back vehicles to choose a safety operational in a connected vehicle environment.

The rest of the paper is constructed as follows. The experiments are designed in Section 2, and the datasets are defined based on the experiments. Section 3 presents the framework of real-time VMDS. Innovated methods are developed in detecting different motions and evaluating the performance. Section 4 provides the performance of VMDS combining mobile sensors. Meanwhile, the benefit of the VMDS in improving driving safety is evaluated. Finally, we conclude the paper and give directions for future work in Section 5.

## 2. Experimental Setup and Dataset

### 2.1. Data Collection by the Android Phone

An Android app is used to collect sensors’ data in the background. Data detected from all kinds of mobile sensors are real-timely processed and transmitted through Wi-Fi Direct. More than 2000 real-world trips’ data, both in urban road and highway, were collected by twenty-three participants.

**Figure 2:**
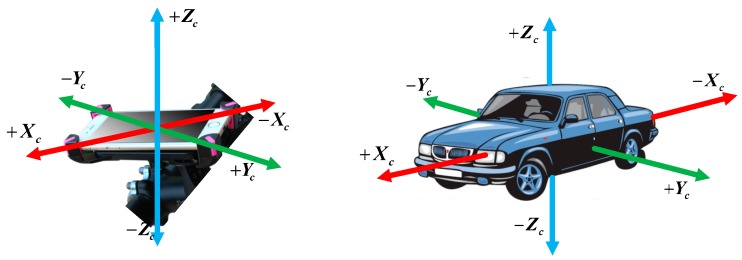
Smart-phone parallel mounted tightly in a vehicle.

There have been many existing research works done using embedded motion sensors for driving behaviors detection [33,34,35]. When a phone (embedded motion sensors) is properly aligned with the vehicle, the gyroscope sensor can infer the vehicle’s lateral dynamics. Similarly, the vehicle’s longitudinal movements can be obtained from accelerometer sensor. Hence, we need to align the smartphone coordinates with the vehicle coordinate before performing the analysis. This allows us to focus on the change of the readings from a single axis for each movement. Coordinate alignment is the process to align the coordinates of the smartphone to those of the vehicles. The Android smart-phone is mounted parallel to the ground level, as shown in Figure 2. We use similar techniques as those mentioned in the work of [36] for coordinate alignment; we refer interested readers to that paper for detailed information.

### 2.2. Distracted Driving and Safety

To explore whether VMDS is helpful for driving safety via changing drivers’ operational strategy, the indoor experiments were conducted with a driving simulator. Participants operated the simulator with input traffic while the app ran in the background. They would be alerted by the phone of the motion changing of the traffic, for example, a lane-changing of front vehicle, a brake of front vehicle, or an acceleration of back vehicle. Voice alert messages are listed as follows:

- Back car accelerating;
- Front car changing lane;
- Front car turning;
- Front car braking.

**Figure 3:**
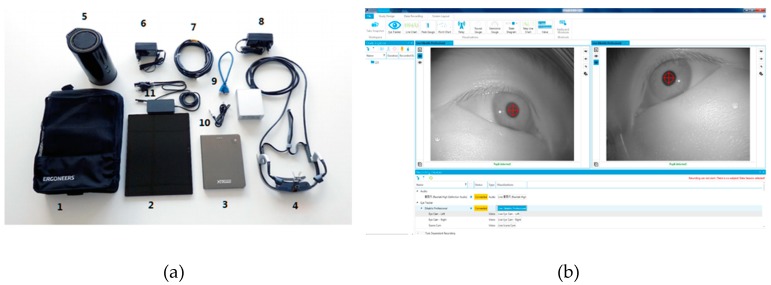
Dikablis Pro eye-tracker (**a**) and D-Lab software user interface (**b**).

**Figure 4:**
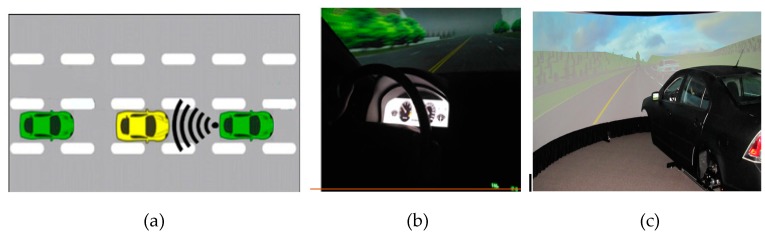
Driving simulator experiment with Wi-Fi Direct (**a**) inside view (**b**) and outside view (**c**).

Participants are set in distracted driving status by man-made distractions like randomly texting, using a smartphone, eating and drinking, talking to passengers, and adjusting the radio. All distractions endanger driver, passenger, and bystander safety. Then, as shown in Figure 3, the Dikablis Pro eye-tracker is used to collect information about drivers’ fixation distribution and changing pupil size [37] with the following configuration: tracking frequency(s) 60 Hz per eye, resolution 648 x 488 pixels, pupil tracking accuracy 0.05° visual angle, glance direction accuracy 0.1°–0.3° visual angle, and field camera aperture angle 40°–90°. Moreover, to avoid the data noise from eye-tracker, participants are selected with qualified eye vision who are able to drive without wearing glasses. D-Lab software is also used for eye movement data ingestion and concurrent processing. Fixation distribution and pupil size can precisely reveal people’s focus and attention. These two variables are used as the index of the driver’s distracted and concentrated state in Figure 4. 

### 2.3. Datasets

In this study, a total of more than 4000 miles’ driving data are collected for training as well as evaluating the VMDS. Our driving data include more than 2137 lane change events and 2972 turn events. The driving data were collected from both highways (state and city highways) and urban roads. Furthermore, our dataset includes data collected during daytime and night hours. It also has driving data collected under different weather conditions, such as sunny, rainy, snowy, and cloudy. In summary, our driving dataset covers most scenarios a driver would face in their daily life. We believe our dataset is good enough to help us build a robust model. We also feel that such a dataset would benefit other researchers in this community, so we plan to open source part of our dataset in the near future (we need some time to go over administrative processes and prepare the data set for public use). Different vehicles using the same phone and mounting set-up are used in this test. The data for VMDS are divided into eight datasets based on road type (urban road and highway) and devices, as shown in Table 1. All of the datasets are collected and processed by Android phones mounted on different vehicles with a customized data collect application running in the background. The Android application records data from the motion sensors (including accelerometer and gyroscope) as well as GPS. The data sampling rate is set to 100 Hz for motion sensors and 1 Hz for GPS. Collected sensor data are stored to an SQLite database locally on the phone. The database files are copied from the phone for further analysis. Although different vehicles and drivers are recruited, only the same phone running the same app is used in a different timeline. The tablets are used to collect the vehicular speed data from various sensor data, including GPS and inertial sensors.

Dataset 1 is the gyroscopes’ sensor datasets. Gyroscopes’ sensor data reflect the angular speed along the *X*-, *Y*-, and *Z*-axis of the phones. These datasets are used to train and test the motion associated with the steering system, like turning and lane changing. For each driver’s data, the datasets are randomly assigned into two parts, 80% data as training datasets and the remaining 20% data as testing datasets (note that the random selection process is based on driving events, that is, 80% of the detected turn event is categorized to training set and 20% is treated as testing set. In other words, the training set includes 80% of detected driving events). Then, different drivers’ training datasets are merged into one large training dataset. The same method is applied to the testing dataset. To avoid over-fitting, k-fold cross validation is also used to randomize the dataset.

In addition, because GPS may lose accuracy to distinguish the lanes of the abreast vehicle, we also use gyroscopes’ data to keep track of lane changing and turning events. The actual lane in which the vehicle is driving can be inferred by combining context information extracted from both GPS and gyroscope sensor data. In general, we use the gyroscope scope as a complimentary mean to determine in which lane the vehicle is driving when GPS suffered a low accuracy (e.g., driving in a tunnel, or in crowded downtown where skyscrapers are nearby).

Dataset 2 is accelerometers’ sensor datasets. In this study, accelerometers’ sensor data are used to estimate the acceleration, deceleration, and brakes.

Dataset 3 represents GPS datasets. A GPS receiver can reliably achieve the 3D localization, time, and speed. In this research, GPS datasets are used to locate the vehicle. 

Dataset 4 shows video datasets. Automobile video data recorder is applied all through the driving tests. The video is replayed as the ground truth recording the start time and end time of each lane-changing and turning.

## 3. Real-Time Vehicle Motion Detection System (VMDS)

### 3.1. Figures, Tables and Schemes

**Figure 5:**
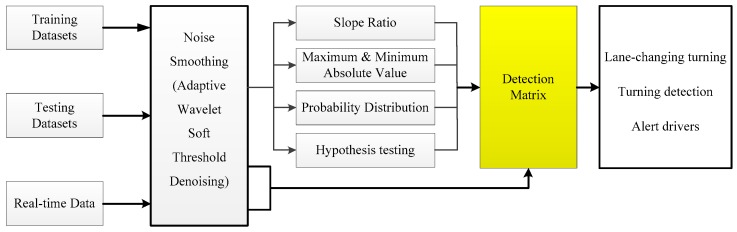
Real-time detecting matrix.

In this section, a novel real-time detecting matrix based on a dynamic time warping (DTW) algorithm with PCA is designed in order to detect lane changing and turning (include curves and intersections), as shown in Figure 5. As is mentioned in the datasets, gyroscopes’ sensor datasets are used for training the motion detecting matrix.

### 3.2. Data Noise Smoothing

**Figure 6:**
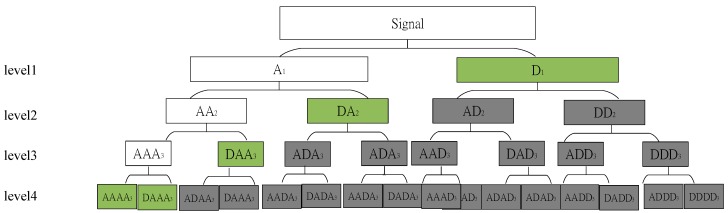
Schema of fast optimal wavelet basis (OWB) algorithm.

As the requirement for computational time is very restrictive for VDMS, we use an adaptive wavelet thresholding function to quickly smooth the noise of the data. An efficient algorithm, that is, the optimal wavelet basis (OWB) noise smoothing method developed by Kaur et al. [38], is selected as the priority step of data processing. As is shown in Figure 6, to choose an optimal basis, fast OWB searches from top to bottom of the tree structure. To calculate the best path, fast OWB accumulates each layer’s value starting from the root to the bottom. Note that it is unnecessary to let the tree grow entirely when generating the optimal basis tree, and thus we use Shannon entropy to create the optimal wavelet basis, as shown in Algorithm 1.

The wavelet packet (WP) of sensor data (time sequence) is applied by a set of filters (band-pass filter). The WP analytic conducts an operation of convolution–decimation. Additionally, this operation recursively occurs on approximation and all the sub-bands. At last, when a large number of possible bases occurs, there are the single best basis representations of the signal offered by WT.

**Algorithm 1:** Fast Optimal Wavelet Basis (OWB) Extraction**Step 1.** Select as the largest levels No. for wavelet packet (WP) dissolution **Step 2.** If the current level (d) of dissolution is smaller than L. Execute 2.1–2.4 to every existent parent-node (sub-band) $S_{d}^{i}(0\leq i\leq 4^{d}-1)$. **Step 2.1** Calculate Shannon entropy $SE(S_{d}^{i})$ of sub-band as the cost function. **Step 2.2** Divide $S_{d}^{i}$ into 4 sub-bands (children-nodes: $LL_{d+1}^{4i}$,$LLH_{d+1}^{4i+1}$,$HL_{d+1}^{4i+2}$ and $HH_{d+1}^{4i+3}$). Calculate their Shannon entropy: ${SE(LL}_{d+1}^{4i})$,$SE(LH_{d+1}^{4i+1})$,$SE(HL_{d+1}^{4i+2})$ and $SE(HH_{d+1}^{4i+3})$. **Step 2.3** If ${SE(S}_{d}^{i}{)<(SE(LL}_{d+1}^{4i}{)+SE(LH}_{d+1}^{4i+1}{)+SE(HL}_{d+1}^{4i+2}{)+SE(HH}_{d+1}^{4i+3}))$ is true, keep the parent node and delete the children-nodes. If it is false, keep both the parent- nodes and children-nodes. **Step 2.4** If no nodes are available to divide, end for extracting the OWB.

### 3.3. Design of Detection Matrix

As the real-time data are collected from multiple sensors on the vehicle, we first describe the overall data resources as well as their unique properties. 

First, accelerometers have high accuracy in estimating accelerations, deceleration, and brakes, especially in high-speed scenarios [39]. The acceleration, deceleration, and brakes are comparatively evaluated by accelerometers data and GPS data. In addition, an accelerometer can be used anytime, even when GPS signal is missing. The acceleration and deceleration results in the following section are mainly detected by accelerometers. 

Inertial measurement unit (IMU) sensors can be found in commercial off the shelf (COTS) devices [40]. They can be used to monitor vehicle dynamics when the sensor is aligned with the vehicle. Gyroscope sensor readings reflect the vehicle’s lateral dynamics, which means turning, lane changing, and swerving events can be inferred using a gyroscope sensor. GPS datasets are used to locate the vehicles’ relative front-back position via reading the geologic information. Because GPS may lose accuracy in distinguishing the lanes of the abreast vehicle, gyroscopes are added to judge the corresponding lane that the vehicle is currently in. The detecting method is based on the assumption that test vehicles are equipped with a smartphone with an app (sensor data collector) running in the background. First, the original lane is recorded after the first turn to the road. Then, each lane change will be detected by gyroscopes and the detecting matrix. Finally, lane status updates in real-time. In this case, the paired relative position can be precisely detected.

After identifying the vehicle motion detections with multiple sensors, we introduce the key idea in designing VMDS. In particular, our aim is to detect in real time lane changing, turning (curve and intersection), and acceleration behaviors using vehicle motion data from multiple sensors. Thus, we especially need to compare the real-timely detect data with a set of standard data, that is, the vehicle motion data under normal states. In other words, we need to compare the real-timely detected data with the standard data set. If a large deviation is observed, for example, a hard brake, VMDS should be able to capture these variations.

**Figure 7:**
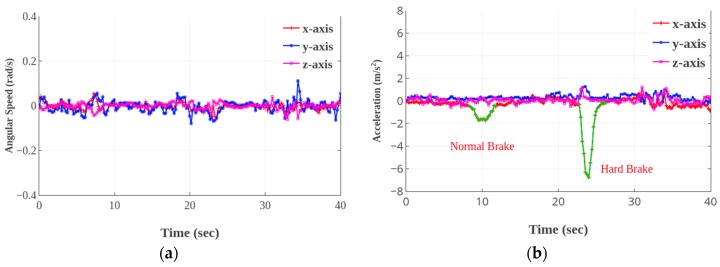
Patterns of brake, angular speed (**a**); acceleration (**b**).

**Figure 8:**
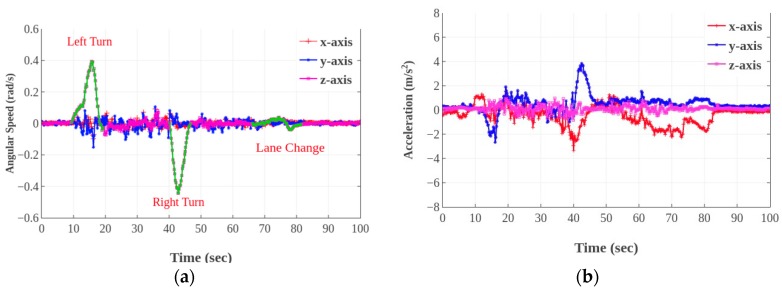
Patterns of lane change and turning, angular speed (**a**); acceleration (**b**).

Therefore, we propose a dynamic time warping (DTW) algorithm under the framework of the dynamic programming technique to measure the similarity between two temporal sequences. It is particularly useful in identifying similar motions with varying speeds or with intermediate accelerations and decelerations. The sequences are ’warped’ non-linearly by shrinking or stretching along the time dimension to determine a measure of their similarity. This is particularly useful in our scenario where different events (e.g., acceleration, brake, turning, and lane changing) may happen at different speeds and the vehicle motions may involve bumps and stops. The core challenge in DTW is in choosing a good training set that is representative of the different types of events. In our scenario, normal and hard brake, various turning angles are two factors that can change between the two events. For illustrative purposes, Figure 7 and Figure 8 show raw accelerometer and gyroscope values for different events. Figure 7 shows a normal brake and a hard brake. Accelerometer readings for the hard brake and the normal brake have the same shape, but different magnitudes owing to a shorter brake time and distance. Figure 8 shows the accelerometer and gyroscope readings when the vehicle heading direction changes, which indicates turn and lane change events. The shapes of the signals, for these driving events, have similar observations (different magnitudes and data shrinks along the time axis). The DTW algorithm and training data set thus need to be adept to deal with such variations.

In order to speed up the data analysis process and detect various driving events in a timely manner, we use principal component analysis (PCA) to reduce the number of variables and extract core features from the collected dataset. PCA eliminates the least important features in the dataset and keeps simplicity and maintains interpretability of your variables [41,42]. Furthermore, PCA combines input variables in a specific way to retain the most valuable features of all of the variables. Thus, we apply the PCA algorithm to our accelerometer and gyroscope dataset and extract core features from them. The following steps summarize the PCA algorithm.

(1) Normalize data

(1) $$X^{*}=X-\mu /\delta ,$$

where *µ* and *σ* are the mean and the standard deviation of all sample data separately, and *X* is a vector that stores the time series data collected from motion sensors. The units of accelerometer and gyroscope data are meter per square second and degree per second, correspondingly. 

(2) Calculate the covariance matrix

(2) $$\mathrm{Matrix}(\mathrm{Covariance})=\left[\begin{array}{ll} \mathrm{Var}\left[X_{1}\right] & \mathrm{Cov}\left[X_{1}{,X}_{2}\right]\\ \mathrm{Cov}\left[X_{2}{,X}_{1}\right] & \mathrm{Var}\left[X_{2}\right] \end{array}\right],$$

where *X_1_* is the time series data collected from accelerometer sensor and *X_2_* is the time series data collected from gyroscope sensor.

Please note that $\mathrm{Var}\left[X_{1}\right]=\mathrm{Cov}\left[X_{1}{,X}_{1}\right]$ and $Var\left[X_{2}\right]=Cov\left[X_{2},X_{2}\right]$.

The classic approach to PCA is to perform the Eigen decomposition on the covariance matrix Matrix(Covariance), which is a d×d matrix where each element represents the covariance between the two features. The covariance between two features is calculated as follows:

(3) $$Cov\left[X_{j},X_{k}\right]=\frac{1}{n-1}\sum_{i=1}^{n}(x_{ij}-\overset{-}{x_{j}})(x_{ik}-\overset{-}{x_{k}}).$$

We can summarize the calculation of the covariance matrix via the following matrix equation:

(4) $$\mathrm{Matrix}(\mathrm{Covariance})=\frac{1}{n-1}\left({(X-\bar{x})}^{T}(X-\bar{x})\right).$$

where $\bar{x}$ is the mean vector $\bar{x}=1/n\sum_{i=1}^{n}x_{i}.$

The mean vector is a *d-*dimensional vector, where each value in this vector represents the sample mean of a feature column in the dataset.

(3) Calculate covariance matrix characteristics

As the covariance matrix is a squire matrix, it is possible to calculate the eigenvalues and eigenvectors for it. We use ƛ to represent the solution of the following characteristic equation (also known as an eigenvalue for a matrix *M*)

det(D*I* − *M*) = 0. (5)

In Equation (5), I is the identity matrix of the same dimension as M. ‘det’ is the determinant of the matrix. For each eigenvalue ƛ, a corresponding eigenvector V can be found by solving the following:

(*I* − *M*)v = 0. (6)

(4) Selecting principal components

The eigenvalues derived from previous steps are ordered from largest to smallest. Sorting them into orders can help us understand the significance of different components. The principal components of the dataset are determined by choosing the highest eigenvalue and its corresponding eigenvector. As there are two variables of the dataset, we can derive two sets of eigenvalues and eigenvectors. We use the feature vector defined in Equation (7) to represent the eigenvector and eigenvalue pairs:

(7) $$\mathrm{Feature}\;\mathrm{Vector}={(V}_{1}{,V}_{2}).$$

(5) Form principle components

As the eigenvectors indicate the direction of the principal components (new axes), we multiply the original data by the eigenvector matrix to re-orient our data onto the new axes. This re-oriented data is called a score.

(8) Sc=[Orig.data]⋅[V]

We build an event library with pre-defined vehicle motion data, which contains all the typical events discussed in previous sections. For each sample event in the library, data collected from both accelerometer and gyroscope are saved into the library. To improve event prediction performance, we included several samples for each event in the library. For example, we include sensor data of normal turn, sharp turn, and turns with different turning radius in our library. New data are then processed using the abovementioned PCA algorithm and compared against all events in the labeled set using the desired temporal sequence, and the distance measure returned by DTW as is shown in **Algorithm 2** is then fed to a k-nearest neighbor algorithm to predict a label for the unseen data.

**Algorithm 2:** Pseudo-code of the algorithm for forming principle components.DTW (_A_, _G_) { // where the vectors A = (a_1_, …, a_n_), G = (g_1_, …, g_m_) are the time series data collected from accelerometer and gyroscope with n and m data points, respectively. Define M [0, …, n, 0, …, m] as a two-dimensional data matrix. It stores the similarity measures between two time series.   / / Data matrix initialization  M [0, 0]: = 0   For i = 0 to m Step 1 Do:    M [0, i]: = Infinity  End  For i: = 1 to n Step 1 Do:    M [i, 0]: = Infinity  End  // Compute the similarity measures between the two time series and store them in M [n,m]  For i :=1 to n Step 1 Do:   For j : =1 to m Step 1 Do:   // Evaluate the similarity of the two points     diff := $d_{mn}(A(i),G(j))$     M [i, j] := diff +Min (M[i-1, j], M [i, j-1], M [i-1, j-1])    End  End  Return M [n, m] }

We tried different metrics to measure the similarity between the two data samples. The metric evaluates the similarity between two data samples by computing the difference between each data point in the sample data. There are four common similarity measurement metrics, the details of which can be found in the work of [43]. We tested our DTW algorithm with the following four similarity measurement metrics, and found out that the symmetric Kullback has the best performance. Hence, we choose the symmetric Kullback to compute the distance between the two data samples.

Euclidean distance is shown as Equation (9):

(9) $$d_{mn}(X,Y)=\sqrt{\sum_{k=1}^{K}\left(x_{k,m}-y_{k,n})*(x_{k,m}-y_{k,n}\right)}.$$

Absolute distance is shown as Equation (10):

(10) $$d_{mn}(X,Y)=\sum_{k=1}^{K}\left|x_{k,m}-y_{k,n}\right|=\sum_{k=1}^{K}\sqrt{\left(x_{k,m}-y_{k,n})*(x_{k,m}-y_{k,n}\right)}.$$

Squared distance is shown as Equation (11):

(11) $$d_{mn}(X,Y)=\sum_{k=1}^{K}\left(x_{k,m}-y_{k,n})*(x_{k,m}-y_{k,n}\right).$$

Symmetric Kullback is shown as Equation (12):

(12) $$d_{mn}(X,Y)=\sum_{k=1}^{K}\left(x_{k,m}-y_{k,n})(logx_{k,m}-\log y_{k,n}\right),$$

where X is the sample data clip of one event in the event library and Y is the data clip of one newly detected event. 

### 3.4. Distracting Driving Evaluation

In this study, drivers’ pupil size is creatively utilized as the corresponding variable with fixation duration. Pupil size and fixation duration can explain whether the drivers are concentrating on driving. Drivers who are driving distracted show smaller pupil size and shorter fixation duration. When they are focusing on traffic or road conditions, their pupil size and fixation duration will increase dramatically.

These two variables, traffic and road condition, are supplemented to each other and enrolled in one function. The percentage of fixation duration is shown as follows:

(13) $$f_{a}=(\sum_{a}^{a-1}t_{x}/\sum_{B}^{A}t_{x})\times 100\%,$$

where $f_{a}$ is the percentage of fixation duration when a−1<x≤a; x is pupil size; $t_{x}$ is the corresponding fixation duration of  x; and A,B is the boundary of pupil size distribution, where A = 24, B = 44.

## 4. Results and Discussion

### 4.1. Lane-Change and Turning Detection

The designed detecting matrix is tested with testing data from No.1 datasets and No.4 datasets. The testing data are inputted incrementally simulating the real traffic and real-time detecting. First, three questions are analyzed as follows.

- When and how accurate the lane-change and turning can be detected?
- Is there a significant influence in lane-change and turning detecting between urban road and highway?
- Is there a significant influence in lane-change and turning detection at a different speed?

**Figure 9:**
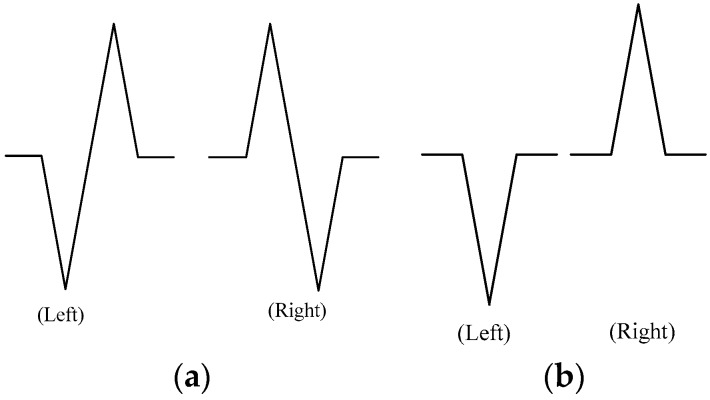
Pattern of lane-change (**a**) and turning (**b**).

To answer the questions, as well as to detect lateral movements or lane-changes performed by the driver, two variables, detecting-time and detecting-accuracy, are applied. As the phone is parallel mounted, only z-axis data of gyroscopes are useful. Using the gyroscopes’ data fluctuation from the lane-change and turning patterns, it is clear to judge lateral movements and distinguish one side lane-change from the other side lane-change. After the training of the detecting matrix, the normal formations of each lane-change or turning maneuver are found, as shown in Figure 9. 

Using the detecting matrix, not only the number of lane-changes and happed time are able to be identified, but also safe or sudden lane-changes and turning can be judged. The unsafe motion would show an extreme result, whose absolute value is over two times that of the normal one. Moreover, the average time to finish an unsafe lane-change is 0.57 times the average time to finish a safe lane-change. The results are added to the detecting matrix as parameters to tag the unsafe motion.

**Figure 10:**
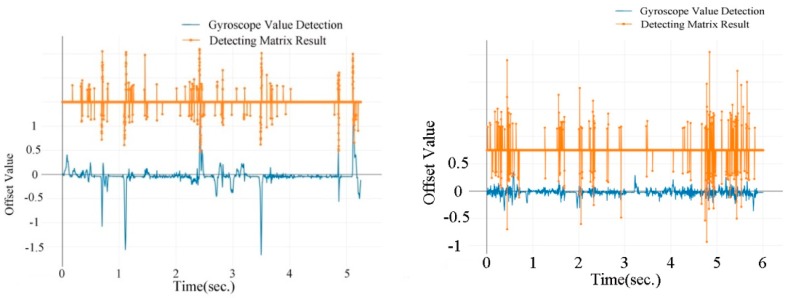
Detection with mobile parallel mounted (**a**) vs. catty-cornered mounted (**b**).

The above analysis is based on gyroscopes data with the phones parallel mounted. Although the Z-axis and Y-axis data have been transferred in the same position as parallel mounted mobile phone, the diverse efficiency is significantly shown in Figure 10. In addition, catty-cornered angles are randomly set in each trace. Particularly, only when the smartphone was parallel mounted to the vehicle, the gyroscopes precisely sensed the movement. Correspondingly, the detecting matrix performed well.

(1) Lane changes

**Figure 11:**
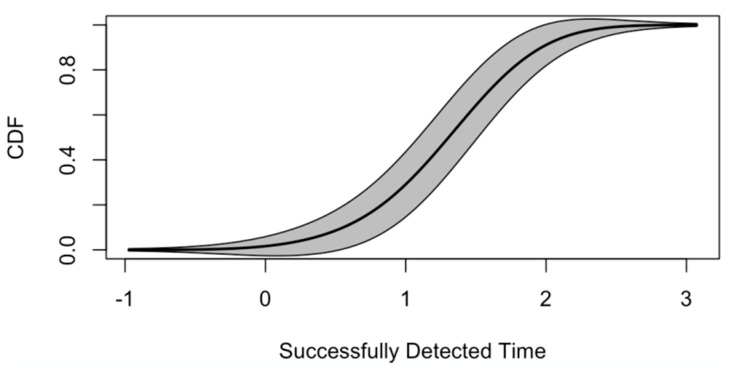
Lane-changes detected time (overall).

The cumulative distribution function (CDF) of time needed (in seconds) for successful lane-change detections is shown in Figure 11. In this study, only lane-changes detected within 2 s from steering wheels moving (delayed period comparing to ground truth) are defined as a successful detection. The thickness stands for the distribution density of detection time. From Figure 11, we can see that nearly 70% of lane-changes are detected within 1 to 2 s. Very few (less than 5%) lane change events require more than 3 s to detect. Most lane-changes are detected within 1.2 or 1.6 s. For human drivers, it usually takes 1.6 to 3.2 s to become aware of the motion of the front vehicle. However, it varies as a result of drivers’ age, vision, fatigued, driving history, and so on [44]. Overall, the VMDS may gain the driver a longer reaction time, ranging from 0.4 to 2 s.

**Figure 12:**
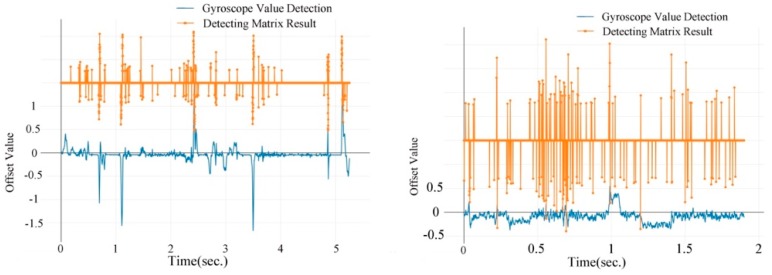
Real-time lane changes recorded by gyroscopes on urban road (**left**) and highway (**right**).

The gyroscopes’ record and detecting results of lane changes are shown in Figure 12. Gyroscopes’ data have less noise on urban road than on highway. As a result, the detecting matrix achieves a better result for urban road than for highway. The noise is caused mainly by poor road conditions, for example, vibrations could be produced by potholes or rugged roads. This will be analyzed in detail later. 

**Figure 13:**
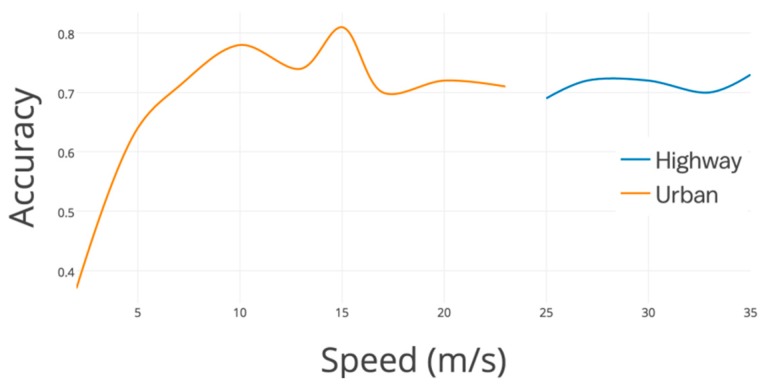
Real-time lane-changes detected accuracy with different speed on urban road and highway.

The performances of lane-changes detection are shown in Figure 13. It needs to be mentioned that, in Figures 13, 15, 18, 19, and 22, the accuracy equals to the right detected event divided by the ground truth. The gyroscope result ranges from 37.5% to 81.2% on urban roads, while it ranges from 68.2% to 72.4% on highway. On the basis of the deviation of these two lines, gyroscope data changes more slightly on highway than urban roads. Although gyroscope data shift with a larger gap on urban roads (with low accurate result), however, the majority of detecting results perform (91.6% data with accuracy above 72% on urban roads) better than that on highway (67.1% data with accuracy above 72%). Especially, when driving between 8 m/s to 16 m/s, the gyroscope result keeps changing slightly, while with high accuracy (from 76.3% to 81.2%). Gyroscope has an unqualified performance when the vehicles just start with a low speed (accuracy is lower than almost 50% with speed lower than 3 m/s), however, low-speed driving barely causes serious accidents. This drawback is not on the high priority list. The results obviously answer the difference between speed distribution on road and highway significantly influence lane-change detecting performance.

Additionally, there are different results of motion detection for vehicles merging at highway/urban on-ramps and off-ramps, as shown in Table 2. Urban off-ramp has the best performance (80.3%), while urban on-ramp has the lowest accuracy (52.6). According to the corresponding speed of each scenario, speed distribution and acceleration/deceleration also affect lane-change detecting performance on urban road, however, they do not obviously affect the performance on highway.

**Figure 14:**
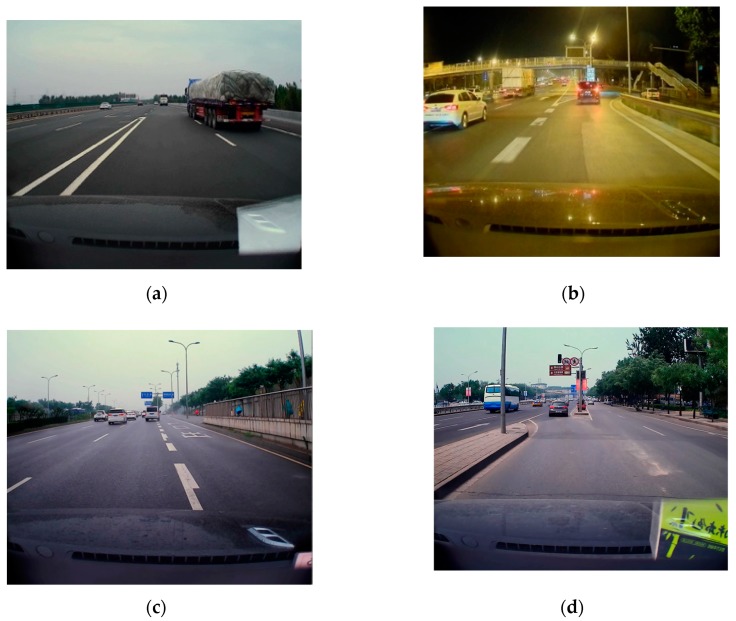
Motion detection for vehicle merging at highway on-ramps (**a**) /off-ramps (**c**) and urban on-ramps (**d**)/off-ramps (**b**).

To explore the reason for the decreasing performance in highway, the detecting accuracy in different road conditions of highway is shown in Figure 14. It is obviously found that the curve and bump both have a strong impact on detecting accuracy and gyroscopes’ efficiency. The detecting accuracy of driving on flat and straight highway ranges from 69.7% to 83.6%, which is much higher than that of driving through the curve (from 58.2% to 67.7%) and on a bumpy road surface (from 51.9% to 62.4%).

(2) Turning 

In this study, turning is integrally identified in terms of steering at the intersection, roundabout, curve, and so on with or without the signal controlled. This detecting matrix can also be utilized to cluster which kind of turning it is, which will be discussed in further specific research.

**Figure 15:**
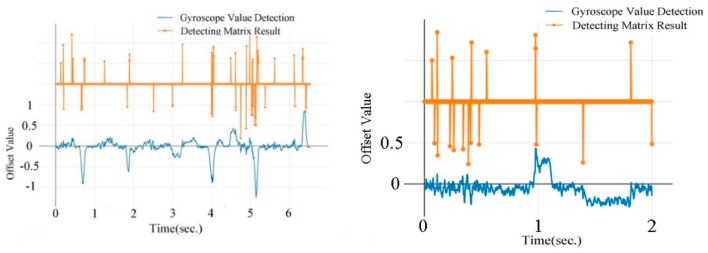
Real-time turning recorded by gyroscopes on urban road (**left**) and highway (**right**).

Figure 15 compares a sample of the gyroscopes and detecting results of turning on urban road and highway. Turing with a small turning circle through urban road happens frequently. However, as a result of high speed, fewer turnings with small turning curves occur during highway trips. Thus, turns are much more easily observed on urban roads than on highway. The majority of recorded turnings happen in urban areas.

**Figure 16:**
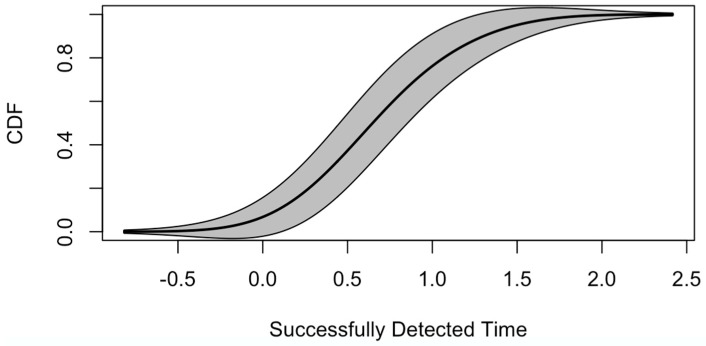
Real-time turning detected time (overall)

Figure 16 shows the CDF of successful turning detections with detected time. In this study, only lane-changes detected within 2 s from wheels moving (delayed period comparing to ground truth) are defined as a successful detection. Almost 80% of turning is detected within 1 second. The most frequent lane-changes detected time is between 0.5 and 1.0 second, while there is always a deceleration before and with turning. Thus, the VMDS may gain the following drivers more reaction time to decelerate.

**Figure 17:**
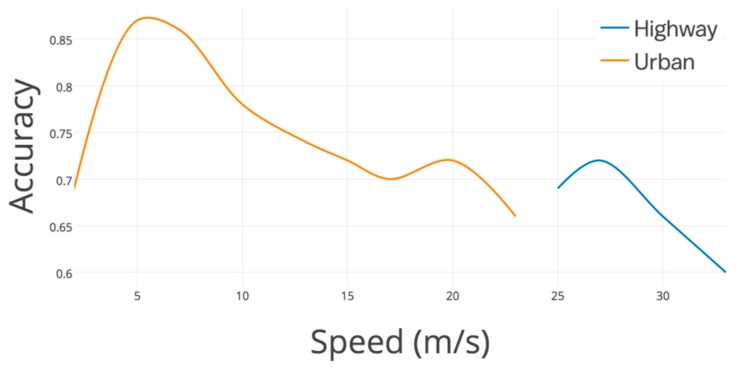
Real-time turning detected accuracy with different speed on urban road and highway.

The results of the turning detected accuracy are shown in Figure 17. It is observed clearly from these two lines that gyroscopes have a great performance in urban areas, especially at a lower speed. However, the detecting accuracy on highway is much worse because of the large turning circle and imperceptibly steering. The detecting accuracy on most urban roads (82.7% data with accuracy above 75%) is better than that of highway (77.1% data with accuracy below 70.0%). In addition, the gyroscopes perform better (above 80.0%) when driving between 3 m/s to 8 m/s. The gyroscopes have some noise data because the vehicles just start at a low speed. However, there is a decreasing trend of accuracy with the speed rising. The results obviously answer that there exists a significant influence in turning detection between urban road and highway, as well as speed distribution.

**Figure 18:**
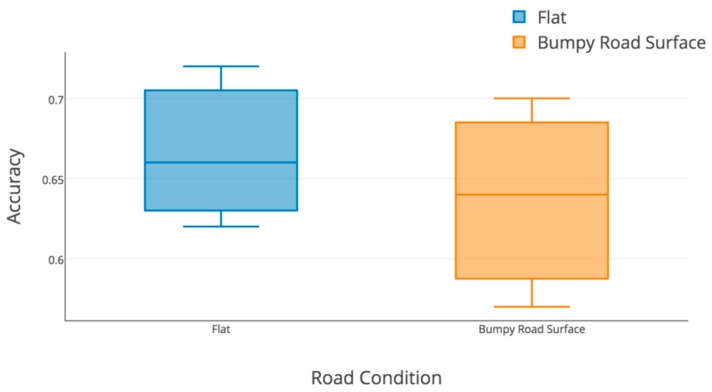
Real-time turning detected accuracy with different road conditions on highway.

As there is a slightly sharp curve in the uncontrolled highway, only the bumpy road surface can impact the accuracy, which is shown in Figure 18. Bumps still decrease the accuracy with a median of 64.2%; the median accuracy of turning detection in flat highway is 66.1%. This gap is not that serious compared with that in lane-change detection. 

Overall, although gyroscopes combined with the detecting matrix are unreliable in some scenarios, the whole detecting accuracy is satisfied, which is better than testing in an instrumented vehicle [45]. The testing detecting matrix results were heavily influenced by the driving environment. There is diverse performance in different traces. However, according to the plenty of real-road data-driven tests, it can be concluded that gyroscopes and VMDS designed in this study can effectively detect lane-changes and turning in real time.

### 4.2. Motion Detection with Accelerometers 

#### 4.2.1. Acceleration Detection

**Figure 19:**
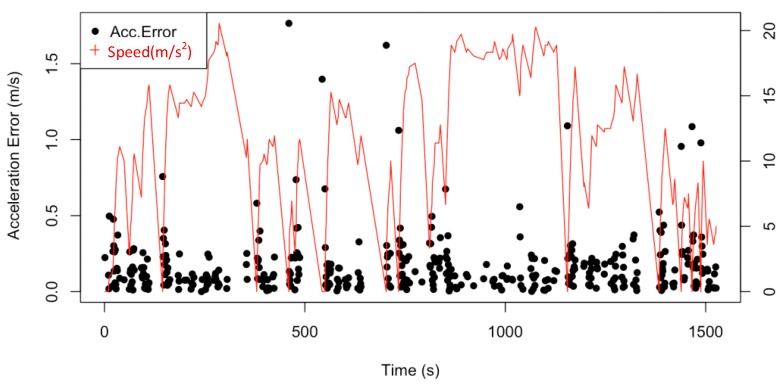
Acceleration detected error being performed with speed.

The acceleration is comparatively evaluated by GPS points and accelerometers’ data. The acceleration detection error is shown in Figure 19. The black points stand for the distribution of the acceleration detection error. The red lines mean the speed corresponding to each acceleration. The estimation errors of accelerometers could be caused by either the bumps or hill road. 

Acceleration detection errors are distributed closer to x-axis when the vehicular speed is higher than 10 m/s. More than 94.7% of the points are able to be accurately detected with an error of less than 0.5 m/s^2^. This result indicates that accelerometers are more accurate in high-speed scenarios. There is another coincide, the singular points appear when the speed is sharply increasing, which reveals that the accelerometers would take time to estimate the fast speed change. However, the errors are mostly between 0 m/s^2^ to 1 m/s^2^, thus the acceleration detecting accuracy is satisfied with driving safety consideration. 

#### 4.2.2. Deceleration and Brakes Detection

**Figure 20:**
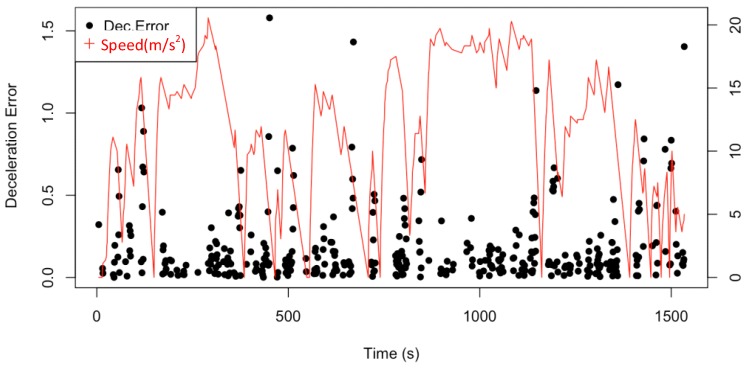
Deceleration and braking detected error being performed with speed.

Generally, deceleration and braking detection errors are slightly larger than acceleration detection errors. As shown in Figure 20, more than 81.4% of the points are able to be accurately detected with an error of less than 0.5 m/s^2^. Similar to acceleration detection, the singular points appear when the speed is sharply decreasing, which confirms that the accelerometers hardly precisely estimate the fast speed decreasing. However, because most of the errors are below 1 m/s^2^, the accelerometers still match the real-time deceleration and braking detection.

### 4.3. Relative Position Detection

**Figure 21:**
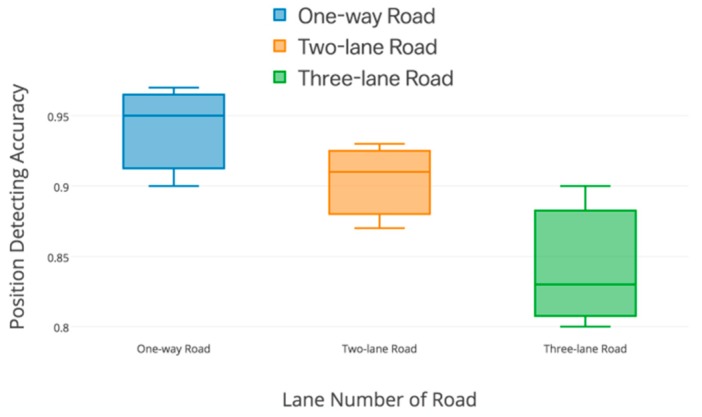
Relative position detection of different roads.

As is shown in Figure 21, the relative position of front-and-back vehicles is precisely judged combining GPS and gyroscopes. In the one-way road, only GPS works achieve great accuracy over 91%. This great result is also observed in the two-lane road with an accuracy of around 90%. Although compared with the accuracy of the one-way and two-lane road, the detection of the three-lane road decreases. The accuracy is still meaningful in alerting the drivers of risks.

### 4.4. Driving Safety and Distraction

**Figure 22:**
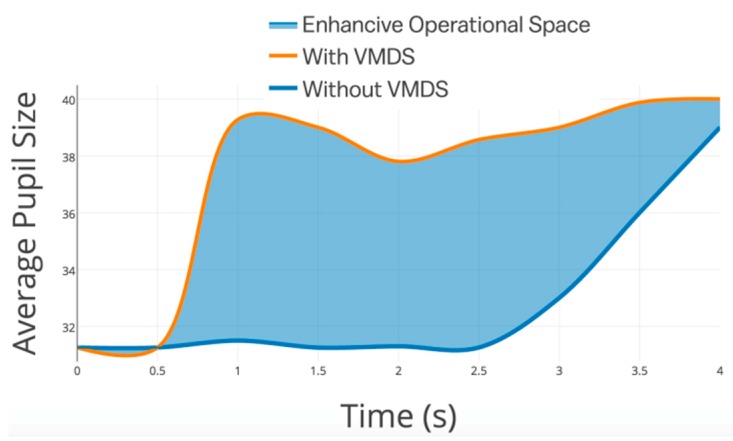
Average pupil size change with time.

Wi-Fi direct is used to transmit self-driving status to other vehicles. In this study, small-scale tests are conducted using simulator vehicles. Several patterns of voice alerts, like front car lane-changing, back car accelerating, front car turning to warn, and so on, are given to participants when they operate the simulator vehicles. Drivers’ behavior and operational changing are recorded with the assistance of VMDS. In addition, fixation duration and pupil size during trips are applied as the index of distraction or concentration. As shown in Figure 22 and Table 3, fixation duration and pupil size of drivers enhanced significantly when the driver started recognizing the potential risks. The average fixation duration increased by 54.2%, while the average pupil size was enhanced by 25.7%. The orange line in Figure 22 stands for the pupil size range with time. Obviously, it gains nearly 2 s more reaction time with VMDS than without VMDS. The blue area means the enhancive operational space, for example, the reacting time and the active braking distance.

The VMDS, as a driver assistance system, aids the driver’s focus back on driving. Thus, the drivers will have more reaction time and operational space in negotiating curves at appropriate speeds, decelerating if an intended lane change may cause a crash with a nearby vehicle, and preparing for an imminent crash. There is a driving safety score for each trip with simulator vehicles. The average score with VMDS is 9.4, which is 2.2 higher than that without VMDS. The innovative VMDS proposed in this study was approved to handle distracted driving, thus improve the driving safety in partly connected vehicles.

## 5. Conclusion and Future Work

Using smartphone devices in term of sensors and Wi-Fi direct, this study developed a real-time VMDS, which aims to capture the traffic motion in the connected vehicle environment in real time and improve driving safety. Considering the computational efforts of smartphone devices and vehicle motion-detecting accuracy, we developed a dynamic time warping (DTW) algorithm with PCA to measure the similarity between two temporal sequences. The similarity with the standard data set can thus present the unexpected motions of a vehicle. VMDS are integrated on Android inside vehicles to evaluate vehicles’ motion and alert the drivers in front/back vehicles.

We conduct field tests to verify the effectiveness of VMDS. Our results indicate that VMDS can achieve high accuracy with real-time computations, making it possible to assist drivers to notice the potential risk earlier both on urban road and highway. Moreover, a Vehicle to Vehicle (V2V) connected vehicle environment is simulated based on the VMDS and Wi-Fi director. Meanwhile, participants are tested via the simulator vehicle; the real-time VMDS was confirmed to alert the driver earlier than without VMDS. In conclusion, real-time VMDS could provide convincing support for the driving safety assistant system in most scenarios. Additionally, IMU sensors are prone to human related activities, such as frequently orientation changes (i.e., frequent phone usage by users) and phone vibrations due to incorrect mounting positions. Hence, it is important to keep the phone in a stable position in order to achieve a better performance.

Mobile phones or smartphones are the most common connection between people and vehicles. In future research, mobile will be explored if it can be used as an embryonic form of the connected vehicle. Thus, the proposed methods need to be more accurate and faster. Also, large-scaled real road tests are necessary to train the more effective motion detection algorithm. In addition, there are several more sensors in smartphones that are expected to be added to motion detection in order to improve the detecting performance.

In summary, with the increasing smartphone penetration level, and the rapid growth of Internet-of-Things (IoT) devices, increasingly more data can be gathered and analyzed to help us gain a better understanding of daily activities. Combining the new techniques in data communication and improved analysis models, we are expecting to find new ways for tackling the prominent risk factors and improving road safety. 

## Figures and Tables

**Table 1:** Datasets.

Road Type	Gyroscopes	Accelerometers	GPS	Video (Ground Truth)
Urban Road	Dataset 1.1	Dataset 2.1	Dataset 3.1	Dataset 4.1
Highway	Dataset 1.2	Dataset 2.2	Dataset 3.2	Dataset 4.2

**Table 2:** Performance of motion detection for vehicle merging ramps

Scenarios	Highway		Urban	
	On-ramp	Off-ramp	On-ramp	Off-ramp
Speed (m/s)	18–33	29–12	2–17	19–7
Accuracy (%)	71.8	69.1	52.6	80.3

**Table 3:** Fixation duration and pupil size facing the potential risk. VMDS, vehicle motion detection system.

	Fixation Duration (Second)		Pupil Size (Pixel)	
	AVG	SD	AVG	SD
Without VMDS	0.334	0.274	31.255	1.371
With VMDS	0.543	0.438	39.276	2.196
